# The Precision of Ophthalmic Calipers: A Potential Reason for Clinical and Surgical Errors

**DOI:** 10.7759/cureus.17438

**Published:** 2021-08-25

**Authors:** Rakan S Al-Essa, Majed S Alkharashi

**Affiliations:** 1 Department of Ophthalmology, College of Medicine, King Saud University, Riyadh, SAU

**Keywords:** caliper, precision, error, measurement, castroviejo

## Abstract

Purpose

The study aimed to determine the precision of different ophthalmic calipers used in our tertiary eye care center.

Methods

All Castroviejo calipers available in the operating room, minor treatment room, and intravitreal injection clinic were included in the study. All calipers were evaluated at four screening points (1, 5, 10, and 15 mm) on a standard ruler and compared to their expected corresponding readings on the caliper scale. If any caliper showed a discrepancy of ≥0.5 mm at any of the screening points, the caliper underwent further analysis on 10 measurement points.

Results

Forty-one calipers were evaluated, of which 16 (39%) showed at least one point of ≥0.5 mm discrepancy on the caliper reading scale. Six calipers had errors >0.5 mm and two calipers had measurement errors of 1 mm in at least one point between 1-15-mm ruler measurement points. The majority of calipers (15/16) overestimated lengths.

Conclusion

Calipers are prone to damage with prolonged use that may affect their precision. Thus, calipers should be calibrated against a standard ruler prior to use even if they look grossly intact. Regular screening of calipers is recommended to identify any discrepancy and prevent avoidable complications.

## Introduction

A caliper is an instrument that measures length. It is used to measure the dimensions of an object or the distance between two points in a plane. Based on their scales, calipers can be classified as either fixed or moveable. Some examples of movable-scale calipers include the Jameson caliper and the Castroviejo caliper. The Jameson caliper is a sliding-type caliper that measures from 0 to 80-mm in 0.5-mm increments. This allows estimates as small as 0.25 mm [1-2]. Moreover, the Castroviejo caliper is the most popular caliber used in ophthalmic procedures because of its small size, ease of use, and precision. It measures from 0 to 20 mm in 1-mm increments, which allows estimates as small as 0.5 mm [3-4]. On the other hand, fixed-scale calipers, such as the Stahl caliper, Braunstein fixed caliper, and scleral ruler, are less commonly used in clinical practice.

Calipers are frequently used instruments in ophthalmic procedures. They have several functions, such as measuring the amount of resection/recession in strabismus surgery, identifying the geometrical center in keratoplasty, planning the site of limbal relaxing incision in refractive surgery, identifying the pars plana in retina surgery and intravitreal injection, diagnosis and follow-up of congenital glaucoma, and measurement of white-to-white corneal diameter for anterior chamber lens implantation [5-7]. Thus, calipers must be accurate to avoid clinical and surgical errors and subsequent complications. The aim of this study was to determine the precision of different ophthalmic calipers used in our tertiary eye care center.

## Materials and methods

This observational study was conducted at the department of ophthalmology, King Abdulaziz University Hospital, King Saud University, Riyadh, Saudi Arabia. All Castroviejo calipers available in the operating room, minor treatment room, and intravitreal injection clinic were included in the study. Prior to enrollment in the study, the calipers were inspected for any damage or missing parts. Calipers then were calibrated against a Codman standard ruler to assess their accuracy.

Initially, all calipers were evaluated at four screening points (1, 5, 10, and 15 mm) on the reference ruler and were compared to their expected corresponding readings on the caliper scale. The four screening points were chosen because most ophthalmic procedures utilize measurements within these ranges. If any caliper showed a discrepancy of ≥0.5 mm at any of the screening points, the caliper was further calibrated against the reference ruler on 10 points (1, 3, 5, 7, 10, 12, 15, 17, and 20 mm). If any caliper showed a discrepancy of ≥0.5 mm at any measurement point, a second testing was performed to confirm the results. Since Castroviejo calipers only have 1-mm increments, the discrepancy was considered 0.5 mm if the point was observed to be visually halfway between two 1-mm increments on the scale. If the point was visually less or more than 0.5 mm but not on the 1 mm-increment on the scale, the measurements were read as 0.25 mm and 0.75 mm, respectively. The amount of error was labeled with the (+) sign if the caliper is overestimating length and the (-) sign if underestimating length. 

Statistical analysis was performed using the Statistical Package for the Social Sciences (SPSS) version 24 (IBM Corp., Armonk, NY). Descriptive statistics were presented using percentages and frequencies.

## Results

A total of 41 calipers were included in this study. Among the 41 calipers, 16 (39%) showed at least 1 point of ≥0.5 mm discrepancy on the caliper reading scale between the 1-15-mm ruler measurements. The error was due to the overestimation of the lengths in most of these calipers (15/16) rather than underestimation. Six calipers had errors >0.5 mm in at least one point between 1-15 mm (Table 1).

**Table 1:** Amount of errors induced by calibers as measured to a standard ruler at each screening point. The (+) sign indicates overestimation

	Ruler								
Caliper	1	3	5	7	10	12	15	17	20
6	+0.50	+0.75	+0.75	+0.75	+0.75	+0.75	+0.75	+0.75	+0.75
7	+0.25	+0.50	+0.50	+0.50	+0.50	+0.75	+0.75	+0.75	+0.75
9	0	+0.25	+0.50	+0.50	+0.75	+0.75	+0.75	+1	+1
17	+0.25	+0.25	+0.50	+0.50	+0.50	+0.50	+0.75	+0.75	+0.75
28	0	0	+0.50	+0.50	+0.50	+0.75	+0.75	+0.75	+0.75
29	+1	+1	+1	+1	+1	+1	+1	+1	+1

Two calipers had errors >0.5 mm at points below 7 mm, and 13 calipers had errors of 0.5 mm below the 5-mm ruler measurement points. Two calipers had measurement errors of 1 mm. It was observed that once a discrepancy was identified at one point, the error either persisted or increased at the subsequent measurement points.

## Discussion

Since the Castroviejo caliper is commonly used in ophthalmic procedures, ensuring the accuracy of these calipers prior to use is critical to avoid multiple clinical and surgical complications such as lens injury or retinal break during pars plana sclerotomy or intravitreal injection, inappropriate corneal graft diameter, inaccurate amount of recession/resection during strabismus surgery, erroneous measurements of corneal diameter in congenital glaucoma, poorly fitted anterior chamber intraocular lens, and improper sizing of limbal relaxing incisions in refractive surgery. This study was conducted to evaluate the precision of the damage-free calipers available in our center that are readily available for use by surgeons/physicians upon request.

In this study, almost 40% of the calipers had at least one point of discrepancy (≥0.5 mm) on the caliper reading scale. Interestingly, 80% of these calipers had errors of 0.5 mm at or below the 5-mm measurement points. These errors underline the risk of critical complications that may occur at such short measuring distances. Moreover, two calipers (13%) had a discrepancy of 1 mm, one of which persistently had this error on all checking points (1-20 mm). This error underscores the risk of harmful clinical and surgical decisions that can result based on erroneous measurements.

Similar to our study, Dahrab and LaRoche assessed all Castroviejo calipers at their hospital. Their results showed that 42% of the calipers had an error of ≥0.5 mm, and 20% of these calipers had a discrepancy of at least 1 mm when calibrated against standard ruler measurement points [8]. Mohamed et al. compared the accuracy of the digital Vernier calipers and the Castroviejo calipers by measuring the horizontal and vertical corneal diameters of human eyes. They reported that digital calipers induced a maximum error of 0.07 mm compared to 0.5 mm by Castroviejo calipers, which is consistent with our findings. The study concluded that Castroviejo caliper measurements are prone to bias and variability compared to those acquired with digital calipers [9]. Moreover, Chen and Osher reported that a 0.1 mm variation was commonly observed when the horizontal diameter of a single cornea was measured by two different Castroviejo calipers [10]. This is consistent with the study published by Mohamed et al., which reported that the precision of manual calipers in ophthalmic biometry measurements is limited to 0.1 mm [9].

Since this study identified a number of calipers with erroneous measurements, we believe that prior to use, any and all calipers must be calibrated against a standard ruler to ensure accuracy. Similar to any instrument, calipers are prone to wear and tear, which may affect their precision. Thus, periodic calibration is necessary to detect imprecise calipers, which can ultimately prevent harmful sequelae. This study did not assess the mechanical reasons causing these calipers to give inaccurate measurements. Further research investigating the mechanical causes will reveal useful information for the early detection of imprecise calipers.

## Conclusions

With prolonged use, calipers may be damaged and worn, leading to inaccurate measurements that may cause serious surgical errors and complications. All calipers should be calibrated against a standard ruler prior to use to ensure precision. It is recommended to conduct regular screening and calibration of all calipers used in surgical procedures to detect inaccuracies and prevent avoidable complications.
